# Acute Liver Failure in an Adolescent Following Use of the Synthetic REV‐ERB Agonist SR9009

**DOI:** 10.1155/crhe/9926772

**Published:** 2026-09-16

**Authors:** Saad Shams Bin Shaheen, Wilson Jing Peng Liu, Caroline Cooper, Michael Yulong Wu

**Affiliations:** ^1^ Department of Gastroenterology and Hepatology, Royal North Shore Hospital, St Leonards, New South Wales, Australia, nsw.gov.au; ^2^ The University of Sydney Northern Clinical School, St Leonards, New South Wales, Australia; ^3^ Department of Anatomical Pathology, Princess Alexandra Hospital, Woolloongabba, Queensland, Australia, health.qld.gov.au; ^4^ Department of Gastroenterology and Hepatology, Bankstown-Lidcombe Hospital, Bankstown, New South Wales, Australia, nsw.gov.au

## Abstract

Drug‐induced liver injury (DILI) is a leading cause of acute liver failure (ALF) and an increasingly recognised consequence of unregulated performance‐enhancing supplements. We report a 17‐year‐old male who presented with progressive jaundice and rapidly evolving ALF temporally associated with ingestion of the synthetic REV‐ERB agonist SR9009. Despite early treatment with N‐acetylcysteine, he developed worsening coagulopathy, hyperbilirubinaemia, ascites and Grade 3 hepatic encephalopathy, necessitating emergency liver transplantation. Extensive investigation excluded viral, autoimmune, metabolic and obstructive causes. Liver biopsy demonstrated severe hepatocellular injury with confluent and multiacinar necrosis, consistent with severe DILI. Following transplantation, liver biochemistry and synthetic function improved rapidly. This case highlights the hepatotoxic potential of unregulated supplements, the importance of obtaining a detailed exposure history in young patients with acute liver injury and the need for early recognition of progressive liver failure requiring timely referral to a liver transplant centre.

## 1. Introduction

Drug‐induced liver injury (DILI) is a major cause of acute liver failure (ALF) in Western countries and remains a common indication for emergency liver transplantation, particularly in patients without pre‐existing liver disease [1, 2]. While paracetamol toxicity accounts for the majority of cases, an increasing proportion of ALF is attributed to herbal and dietary supplements, many of which are not subject to regulatory oversight [3, 4].

SR9009 is a synthetic agonist of the nuclear receptor REV‐ERBα, initially developed as a research compound to study circadian rhythm and metabolic regulation. Despite lacking regulatory approval for human use, it is widely marketed online as a performance‐enhancing supplement. Human safety data are extremely limited and its hepatotoxic potential has not been systematically evaluated. We describe a case of ALF in an adolescent following SR9009 ingestion that progressed to emergency liver transplantation.

## 2. Case Presentation

A previously healthy 17‐year‐old male presented with a 1‐week history of progressive jaundice, dark urine, lethargy, and right upper quadrant abdominal pain. Approximately three months before presentation, he had completed an eight‐week course of orally administered SR9009, which he had purchased online for bodybuilding purposes. He denied alcohol misuse, paracetamol overdose, use of other recreational drugs or supplements, recent travel or high‐risk sexual exposures. There was no personal or family history of liver disease. The chronology of SR9009 exposure, clinical presentation, investigation and management is summarised in Table 1.

**TABLE 1:** Chronological clinical course.

Timepoint	Clinical course
SR9009 exposure	Completed an approximately 8‐week course of orally administered SR9009.
∼3 months later	Developed progressive jaundice, dark urine, lethargy and right upper quadrant pain.
Presentation	Severe hepatocellular liver injury with impaired synthetic function. Extensive investigations commenced.
Week 1 of admission	Alternative aetiologies excluded. Liver biopsy confirmed severe drug‐induced liver injury. Intravenous N‐acetylcysteine continued.
Week 2 of admission	Progressive hyperbilirubinaemia, worsening coagulopathy, ascites and grade 3 hepatic encephalopathy despite supportive therapy.
Shortly thereafter	Emergency orthotopic liver transplantation.
Recovery	Rapid improvement in liver function following transplantation and discharge home on immunosuppressive therapy.

*Note:* Chronological summary of the patient’s clinical course from SR9009 exposure to liver transplantation and recovery.

On presentation, he was haemodynamically stable and alert without evidence of hepatic encephalopathy. Physical examination demonstrated marked jaundice and right upper quadrant tenderness without features of chronic liver disease. Initial laboratory investigations demonstrated severe hepatocellular liver injury with alanine aminotransferase (ALT) 2364 U/L, aspartate aminotransferase (AST) 1132 U/L, total bilirubin 171 μmol/L and an international normalised ratio (INR) of 1.7. Over the following week, liver biochemistry and synthetic function progressively deteriorated despite supportive management, with peak bilirubin of 557 μmol/L, ALT 3178 U/L, AST 1834 U/L and INR 3.4. Serial laboratory investigations are summarised in Table 2.

**TABLE 2:** Serial laboratory investigations demonstrating the evolution of ALF.

Laboratory parameter	Presentation	Peak injury (1‐week post presentation)	Pretransplant	Discharge
Total bilirubin (μmol/L)	157	335	402	26
ALT (U/L)	3928	4204	1870	137
AST (U/L)	2727	3492	1330	32
ALP (U/L)	209	207	237	136
GGT (U/L)	277	172	92	269
INR	2.0	3.0	3.8	Normalised^∗^
Albumin (g/L)	35	30	26	24
Platelet count (× 10^9^/L)	210	205	195	200

*Note:* ALT, alanine aminotransferase; ALP, alkaline phosphatase; AST, aspartate aminotransferase; LDH, lactate dehydrogenase.

Abbreviations: GGT, gamma‐glutamyl transferase; INR, international normalised ratio.

^∗^INR had normalised by discharge according to the clinical course.

Extensive investigations for alternative causes of acute liver injury were unrevealing. Serological testing for hepatitis A, B, C and E viruses, Epstein–Barr virus, cytomegalovirus and human immunodeficiency virus was negative. Autoimmune liver serology, including antinuclear antibody, antismooth muscle antibody and antiliver kidney microsomal antibody, was negative, with normal immunoglobulin G concentrations. Copper studies and MRI‐Brain demonstrated no evidence of Wilson disease. Abdominal ultrasound and magnetic resonance cholangiopancreatography revealed no biliary obstruction or vascular abnormality.

**FIGURE 1:**
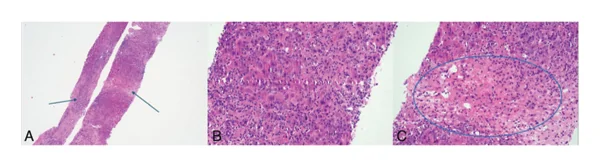
Histopathological features of liver biopsy showing severe hepatocellular injury with confluent and multiacinar necrosis, prominent bile ductular reaction and collapse of the reticulin framework (haematoxylin and eosin stain). (A) Low‐power view demonstrating broad areas of confluent necrosis with hepatocyte dropout (arrows) (× 40). (B) High‐power view demonstrating lobular disarray with associated inflammatory infiltrate (× 200). (C) High‐power view demonstrating extensive pericentral necrosis in Zone 3 (circle) (× 200).

As liver function continued to deteriorate despite supportive management, a percutaneous liver biopsy was performed. Histopathological examination demonstrated severe acute hepatocellular injury characterised by confluent and multiacinar necrosis with extensive hepatocyte dropout, cholestasis, bile ductular reaction and reticulin collapse, consistent with severe DILI. There was no histological evidence of chronic liver disease, autoimmune hepatitis or another underlying hepatic disorder (Figure 1).

The patient was commenced on intravenous N‐acetylcysteine and supportive management; however, his liver function and clinical status continued to deteriorate. He developed worsening jaundice, progressive coagulopathy, ascites and Grade 3 hepatic encephalopathy, consistent with ALF. Immediately prior to transplantation, laboratory investigations demonstrated a total bilirubin of 535 μmol/L, ALT 1519 U/L, AST 344 U/L and an INR of 5.5 (Table 2). Given the development of ALF with progressive synthetic dysfunction and Grade 3 hepatic encephalopathy despite maximal supportive therapy, he underwent emergency orthotopic liver transplantation.

The postoperative course was uncomplicated. Liver biochemistry and synthetic function improved rapidly following transplantation, and he was discharged home on standard immunosuppressive therapy and remained well on outpatient hepatology follow‐up.

## 3. Discussion

This case describes ALF in an adolescent temporally associated with ingestion of SR9009, an unregulated synthetic REV‐ERB agonist marketed as a performance‐enhancing substance. The clinical course, exclusion of alternative aetiologies and characteristic histopathological findings strongly support a diagnosis of severe DILI temporally associated with SR9009 exposure [4].

The differential diagnosis of ALF in adolescents is broad and includes viral hepatitis, autoimmune hepatitis, Wilson disease, metabolic disorders and ischaemic hepatitis [1, 2]. In this patient, extensive investigations excluded these competing diagnoses. Serological testing was negative for viral hepatitis and other infectious causes, autoimmune screening was unremarkable, copper studies and MRI‐Brain demonstrated no evidence of Wilson disease and liver histology demonstrated no features suggestive of autoimmune hepatitis or alternative chronic liver disease. The close temporal relationship between SR9009 exposure and symptom onset, together with the exclusion of alternative causes and biopsy findings demonstrating severe hepatocellular injury with confluent and multiacinar necrosis, strongly supports SR9009‐induced DILI as the most likely diagnosis.

Herbal and dietary supplement–associated liver injury is an increasingly recognised cause of ALF, particularly among young individuals without pre‐existing liver disease [3]. The growing popularity of performance‐enhancing substances has introduced increasing exposure to synthetic compounds with limited or absent human safety data [3, 4]. Unlike licenced pharmaceuticals, these products are frequently manufactured outside regulated pharmaceutical pathways and their composition, purity and dosing may vary considerably, increasing the potential for unpredictable toxicity [4].

SR9009 is a synthetic agonist of the nuclear receptor REV‐ERBα, originally developed as an experimental compound for research into circadian rhythm and metabolic regulation [5]. Experimental studies suggest that REV‐ERBα influences hepatic lipid metabolism, mitochondrial function and inflammatory signalling, providing biological plausibility for hepatic injury following exposure to SR9009 or related compounds [5]. However, SR9009 has never undergone clinical evaluation in humans and there are currently no published data regarding its pharmacokinetics, long‐term safety or hepatotoxic potential [5]. Consequently, the mechanisms underlying hepatotoxicity remain uncertain although direct hepatocellular toxicity, toxic metabolites or variability in supplement formulation may all contribute.

The increasing availability of unregulated performance–enhancing substances poses an important diagnostic challenge for clinicians. Because these products are widely available online and are often perceived to be safe, patients may not volunteer their use unless specifically asked. A targeted exposure history that specifically includes over the counter medications, performance‐enhancing substances and experimental research chemicals may facilitate earlier diagnosis, prompt withdrawal of the offending substance and timely referral to specialist hepatology services [3, 4].

The patient received intravenous N‐acetylcysteine, which has been shown to improve transplant free survival in select patients with nonparacetamol ALF [6]. Despite supportive therapy, he developed progressive hepatic decompensation with worsening coagulopathy, hyperbilirubinaemia, ascites and Grade 3 hepatic encephalopathy, necessitating emergency liver transplantation, after which liver function recovered rapidly.

This case highlights three important clinical lessons. First, unregulated performance–enhancing substances such as SR9009 should be recognised as potential causes of severe DILI and ALF. Second, a comprehensive history of supplement use, including performance enhancing substances and experimental research chemicals, is essential when evaluating young patients presenting with unexplained acute liver injury. Finally, the progression of ALF should be assessed using the overall clinical picture, including hepatic synthetic function and encephalopathy, rather than aminotransferase concentrations alone, as liver enzyme levels may decline despite worsening hepatic failure [7].

To our knowledge, this is the first published report of ALF requiring liver transplantation temporally associated with SR9009 ingestion.

## 4. Conclusion

SR9009 should be recognised as a potential cause of severe DILI and ALF. This case highlights the importance of obtaining a comprehensive history of supplement and performance‐enhancing substance use in patients presenting with acute liver injury, particularly when routine investigations fail to identify an alternative cause. Given the increasing availability of unregulated synthetic compounds, clinicians should maintain a high index of suspicion for supplement‐associated hepatotoxicity. Early referral to liver transplant centres remains essential for patients with progressive hepatic dysfunction despite supportive management.

## Funding

This research received no specific grant from any funding agency in the public, commercial, or not‐for‐profit sectors. The work was conducted as part of the authors’ clinical and academic duties. Open access publishing facilitated by The University of Sydney, as part of the Wiley ‐ The University of Sydney agreement via the Council of Australasian University Librarians.

## Consent

Written informed consent for publication of the clinical details and associated images was obtained from the patient and their legal guardian prior to submission.

## Conflicts of Interest

The authors declare no conflicts of interest.

## Data Availability

The data supporting the findings of this study are included within the article. No additional datasets were generated or analysed during the current study.
